# Metropolitan Medical Schools

**Published:** 1893-09-16

**Authors:** 


### The Medical Student's Vade Mecum.

### THE METROPOLITAN MEDICAL SCHOOLS.

Some Results of a Personal Inquiry.

The parent who has made up his mind to send his son to London to be educated for the medical profession has often very great difficulty in deciding between the conflicting claims of the eleven medical schools which the Metropolis possesses. Each of the eleven enjoys a good reputation. There is not one of them which has not one or more names of note on its staff list. Each makes provision for the teaching of every conceivable subject within the range of medical study.

### How to Find the Best School.

How, then, is the parent, who has never given a serious thought to their respective claims until now, when the matter is forced upon him in connection with the life prospects of a son, to make a choice? A study of the prospectuses of the different schools will not avail him much. What the puzzled father usually does is to consult someone in whose judgment he can trust—as often as not his own doctor. The doctor knows best the school at which he was himself educated; he believes in it, he is loyal to it, and he naturally recommends it. We ourselves have frequently been consulted on this matter, and we are in a position fully to sympathize with parents who are in doubt with regard to it. But while we are always glad to give what assistance is in our power, we feel that it would be very much better if parents were put in a position to make the choice for themselves. One cannot give really useful advice as to a medical school without knowing a good deal of the young man in regard to whom advice is sought—about his disposition, his previous education, his views and the views of his parents as to his future in the profession, and, it may be added, of the financial circumstances of his parents. If one knew all these things one might give advice with less hesitancy, for the truth is that, in London at any rate, one medical school differs from another not so much absolutely as comparatively. One school is strong on one side, another on another. One school has a reputation for the clinical teaching in its hospital, another for the systematic teaching in its class-rooms. One has the best dissecting room in London, another the best physiological laboratory. The school that may be best suited for the youth whose early education has not been over liberal, who after he has become qualified will have to rough it in building up a practice for himself, may not be equally suitable for the graduate of Oxford or Cambridge, who aspires to a University degree in medicine, and who, if all goes well, is not likely to have much concern as to ways and means. The question is not, Which is the best medical school in London? but, Which is the best school in the particular case? What the parent is called upon to decide is at which of the schools his views as to the future of his son can best be met, and in order that he may make this decision aright he must have some information as to the distinctive features of each.

### A Suggestion for Managers.

A real service would be done if someone who knows about the London schools would set forth impartially wherein the strength of each lies. Meanwhile, it may be suggested that it would be a good plan for each school to charge a small committee, composed of elderly men on the staff—men in point of years and feeling in touch with the parents who seek information and advice—with the duty of taking counsel with those who have sons that are about to become medical students. An ignorance prevails as to the work of the medical schools for which the schools themselves are in the main responsible. It was in the hope of being able in some small measure to dispel this ignorance, and of eliciting some facts that might be useful to perplexed parents, that a Commissioner of The Hospital recently made an effort, by means of visits to the schools, of personal interview, and otherwise, to ascertain wherein it is that one London medical school differs from another. That this task was made much easier through the kindness of deans, of members of the various staffs, and of the secretaries of the several schools, must here be gratefully acknowledged.

### The Eleven Schools.

Not to include at present the London School of Medicine for Women, there are eleven hospitals in London which have medical schools. These eleven schools may conveniently be divided into three classes. There are, in the first place, the schools attached to the four great East and South London hospitals—St. Bartholomew's, Guy's, St. Thomas's, and the London. In respect of their age, and of the size of the hospitals with which they are connected, these occupy a place of their own in the mind of the public. The importance in connection with a medical school of ample opportunities for clinical instruction is not to be overestimated, and the very fact that these hospitals are amongst the largest in the kingdom—the London is the largest—is in itself sufficient to invest the medical schools that are attached to them with a peculiar prestige. King's College and University College form a second class by themselves, inasmuch as they are educational institutions on a large scale, which have arts and science as well as medical faculties, and although they have not the right of conferring degrees, they approach nearer to Universities than any other of the London schools. The remaining five schools constitute a third group. The hospitals to which they are attached are smaller than those in the first class. This is not said to their disparagement. In fact, some of these latter schools are at present in a most thriving condition, and every one of them is healthy.

### Guy's Hospital.

As it happened, Guy's Hospital School was the first to engage the attention of our Commissioner. Guy's is one of the four large hospitals that have been mentioned, and it was fitting enough that it should come first, for this school may fairly be regarded as a typical London medical school. Guy's is the hospital for the south-east of London, and it is situated in the busy and densely-populated Borough. Careful parents need have no apprehensions on the ground of the site of the hospital, for no one who knows the situation and the buildings would ever dream of the student there coming by physical or moral harm from his surroundings. One cannot visit Guy's Hospital without realising that the authorities have spared no pains to maintain the school at a high standard, and to promote the well-being of the student. Guy's has kept pace with the times. Of late years much greater stress has been laid on the scientific side of medical education. Physics, biology, and bacteriology have taken their place along with anatomy, physiology, and pathology. All these additions to the curriculum have produced corresponding changes and additions in the classes and buildings at Guy's. The substantial block named the Petersham Buildings, that was recently completed, bears ample evidence to the desire of the authorities to keep the school thoroughly up-to-date. This building is mainly devoted to the needs of the dental school—for Guy's is alone among the general hospitals of London in having attached to it a fully-equipped dental school; and it also contains a chemical laboratory, which has places for a hundred students, a lecture-room, and laboratories for experimental physics and bacteriology. Guy's Hospital itself, with over 500 beds in constant occupation, affords ample clinical material. The midwifery department is very large; it is claimed for the lying-in charity of the hospital that it is the largest connected with any English school of medicine. The number of cases attended by the extern obstetric assistants last year was 3,144.

The authorities at Guy's have not forgotten to care for the well-being of the student. The home-like character of the school could not fail to impress itself on the visitor. Guy's has a residential college which accommodates sixty students. The college building, which was opened in 1890, has been planned to serve the double purpose of a residential college and of a home for the students' club. The dining hall, the library, the gymnasium, the smoking-room are large, well lighted, and well aired. The sets of rooms that are set apart as sitting-rooms and bed-rooms for students are simply but comfortably furnished. The popularity of the college is sufficiently attested by the fact that all the rooms have been engaged for the ensuing session. Some make it an objection to residential colleges that when a large company of young men live together under one roof they are apt to give to conversation and recreation time that ought to be given to study. But in the college at Guy's every care is taken to discourage trifling. The gentlemen who compose the resident staff of the hospital have their rooms in the college which is connected by a subway with the hospital, and in this way the juniors have the advantage of the example and the society of their seniors. Moreover, all the resident students are under the supervision of a resident warden. The terms for board and rooms are very reasonable. Board may be had at a guinea per week, and rooms at charges ranging from 9s. to 20s. per week. At Guy's, as at most of the London hospitals, the various clubs connected with the school have been amalgamated into a clubs' union. By the payment of a composition fee to this union, any student of the hospital can obtain the privilege of membership of all the constituent institutions. The The clubs' union has acquired a recreation park of nine acres in extent, close to Honor Oak Park Station, within easy reach of the hospital. It is worthy of mention that the school at Guy's has a journal of its own in the *Guy's Hospital Gazette*. This well-conducted periodical recently celebrated its majority, and it is in the proud position of being the oldest medical students' journal in the world. It is published fortnightly.

To speak generally, it may be said of Guy's that a thoughtful care for the physical and moral welfare of the students goes hand in hand with an intelligent zeal for the character of the school as a centre of medical education.

### St. Bartholomew's.

A good deal of space has been devoted to Guy's because that school illustrates several of the leading features of the London schools as a whole. The next school visited by our Commissioner was that attached to St. Bartholomew's Hospital. The hospital of St. Bartholomew's was founded in 1123, and there are records of a medical school connected with the hospital as early as 1662. The school at St. Bartholomew's is thus the oldest medical school in London. It is attached to an hospital that is the second largest of the metropolitan hospitals, and the wealthiest of all. The hospital has been so well organised that although the number of students at the school is very large, the most is made of the clinical opportunities. The appointments open to students at the hospital are very numerous. All the London medical schools are able to offer prizes in the shape of scholarships and exhibitions; in this respect St. Bartholomew's is exceptionally rich. In addition to all these things, the authorities have spared neither pains nor money in equipping every department with singular completeness. The school buildings are comparatively new. Replacing old buildings that had been found too small, they were erected at a cost of £50,000, and were completed in 1881. They include an admirably fitted-up set of laboratories, and a dissecting room which is one of the finest, if not the finest, in London. The museums are a special feature of St. Bartholomew's. For the pathological museum it is claimed that it is second only to the Pathological Museum at Berlin. There is a residential college at St. Bartholomew's. The various clubs at St. Bartholomew's were amalgamated into a students' union last year, and negotiations are at present on foot for the lease or purchase of a recreation park, which is expected to be ready next May. St. Bartholomew's is about to emulate Guy's in the field of journalism. It is announced that a St. Bartholomew's hospital journal, to be published monthly, will be started in October.

It is not surprising that a school which enjoys all the advantages that are enjoyed by St. Bartholomew's has become popular. In fact, the danger that St. Bartholomew's must be careful to avoid is that of allowing the school to become too large. The opportunities for clinical and practical instruction that are at present afforded at the hospital and school are splendid; but there is a limit to the usefulness of even these exceptional facilities, and in the case of a medical school that is so fortunate in every way as St. Bartholomew's, it is both proper and needful that this should be pointed out.

### St. Thomas's.

It may be said with little fear of contradiction that there is no hospital in the world which occupies a finer site than the hospital of St. Thomas. The governors of St. Thomas's Hospital and Medical School have good right to be proud of their buildings. But St. Thomas's has greater attractions to offer the aspiring physician and surgeon than fresh air and a magnificent view, albeit these are not to be despised. The present buildings of St. Thomas's were erected at a more recent date than those of any other general hospital in London. In respect of construction, this hospital as the newest is also the best planned of the four great hospitals; and this is of importance from the point of view of the medical student as well as from that of the patient. To walk the wards of an hospital like St. Thomas's is in itself an education. There is an operation theatre in the hospital, which has recently been renovated according to the latest ideas as to operation theatres. This theatre is said to be now one of the finest in the world. In it the risks of septic poisoning have been reduced to the very minimum. The block of St. Thomas's which has been devoted to the medical school is at the southern extremity of the hospital, between Lambeth Palace and the Thames. The school buildings are not large, but two wings are being erected to provide much needed accommodation. In these additions there will be room for a students' club-room and for a pathological laboratory. Each department is by itself, and room has been found not only for the medical classes proper, but for science classes also, at which the student is prepared for the preliminary examination in science of the University of London. The St. Thomas's museum is one of the best in London. The teaching, both practical and systematic at St. Thomas's, is sound and thorough, and excellent results have been attained. The clinical teaching is particularly good, in every way worthy of the reputation of the hospital. There is no residential college, but students have no difficulty in obtaining suitable lodgings within easy reach of the school.

### The London Hospital.

The most noteworthy feature in connection with the London Hospital is that it has a larger number of beds than any other hospital in this country. That an hospital should contain 776 beds is a fact of the first importance in regard to the clinical teaching at the school. The field of the London Hospital is practically the whole of the East-end, and in view of this it may fairly be held to be the largest accident hospital in the world. Parents hesitate about sending their sons to Whitechapel; but let anyone who has a prejudice against the London School, because it is in the East-end, pay the school and hospital a visit. The buildings occupy extensive grounds in Whitechapel Road, which is in more than one respect one of the finest streets in London. The school is within easy access by several lines of railway of all parts of the metropolis. In his journey to and from the hospital, the student incurs no risk that is not common to most parts of London. In short, the objections to the London Medical School on the ground of its situation are hardly serious. Any disadvantages in this respect are amply counterbalanced by the magnificent opportunities that the hospital affords for clinical study. These can hardly be exaggerated. The number of appointments is very large, and the student may always have his hands full of work in the wards. "The London Hospital enjoys the credit of having, in 1785, set the first example of a complete medical school in connection with an hospital upon the model of the medical faculty of a university." The school has all along occupied a large place in the minds of the Governors, and its interests have been steadily promoted. The present buildings, which are new, are commodious, well-arranged, and suitably fitted up. There is a roomy, well-lighted dissecting-room. The anatomical and pathological museums are extensive and well arranged, the latter being particularly rich in specimens, and special mention must be made of the library, a spacious and lofty hall, which is justly admired by all who visit the school. There is no residential college, but there is a students' club, which, generously aided by the members of the House Committee and the staff, has been able to make provision in the new buildings for students obtaining a comfortable meal at a reasonable cost, and with little loss of time. The various school societies have amalgamated to form a clubs' union, which has its own grounds at Lower Edmonton.

These four medical schools—Guy's, St. Bartholomew's, St. Thomas's, and the London—have several features in common, which are determined by their connection with the four largest metropolitan hospitals, but each school has its own distinct individuality, nevertheless. Guy's has its reputation for healthy discipline, combined with excellent teaching; St. Bartholomew's has the prestige which comes of its age and its wealth, and its model arrangements for practical work of every kind; St. Thomas's has its unrivalled situation, and its hospital, in which almost every requirement as to hospitals, has been met; and the London has its magnificent opportunities for clinical study.

A word as to the cost of medical education at these four schools. The question of fees may be disposed of very briefly by setting down the amount of the composition fee, covering all lectures and hospital practice, at the schools that have been named. The fees charged are as follow:—

| | |
|---|---|
| St. Bartholomew's ... ... ... | £157 10s. |
| Guy's ... ... ... ... ... ... | 150 |
| St. Thomas's ... ... ... ... | 150 |
| London ... ... ... ... ... | 120 |

In the three first the fees are substantially the same. At the London they are considerably lower; moreover the London intimates that in the case of sons of medical men a special reduction of fifteen guineas is made.

### University and King's Colleges and their Medical Schools.

The medical schools at University College and King's College have the distinction of constituting medical faculties of fully organised colleges, which have their faculties of arts and of science, as well as of medicine. Wherein is this to their advantage as medical schools, it may be asked. In this chiefly, that the facilities which are afforded for acquiring a sound general education as well as the atmosphere of culture, which it may be taken for granted prevails at these colleges, are calculated to have a broadening effect upon the medical student, to remind him that there are more things in heaven and earth than are included within the five years' curriculum. Moreover, the teaching of those sciences that have their place in the medical curriculum, but are not strictly medical, is delivered from the narrow utilitarian spirit. Something is attained of the breadth of view and general culture that characterise or ought to characterise the University.

### University College Hospital.

In the imposing buildings in Gower Street, where University College has its home, medicine and the sciences have got their due share of room. On the opposite side of Gower Street is the College Hospital, founded in 1833, with 207 beds—a somewhat small hospital in view of the importance and reputation of the school. That reputation is well deserved, for University College has rendered signal service in the cause of reform in medical education. Some facts in the history of the school, which we have on the excellent authority of Mr. Erichsen, the eminent surgeon who worthily occupies the position of President of the College, deserve to be re-stated, because they have been somewhat lost sight of. When University College was founded 70 years ago it was quite common in the London schools for the same man to be lecturer in several subjects. University College led the way in putting an end to this pluralism. University College set the example in London of giving prizes and certificates in the various classes. It was the first school in England at which a separate chair of physiology was established, and the first to set up a chair of pathological anatomy. Not many years ago it was the practice in the metropolitan hospitals for members of the staff to derive a revenue from fees paid by students for hospital appointments. University College Hospital was the first to throw all these appointments open to competition. The roll of men of distinction who have been connected with the College is long and brilliant. It includes the names of Liston, of Syme, of William Sharpey, who was the first professor of physiology in the College; and of Robert Carswell, the pathologist, whose unique pathological drawings are carefully preserved in University College library. These things and these men belong to the past, but a school that has such a history is not likely to fall into decadence. University College shows no signs of falling off. As might be expected, a large number of University College men enter for the degrees of the University of London, with what success may be inferred when it is stated that the gold medal in connection with the M.D. examination has been carried off by University College oftener than by any other school.

### King's College Hospital.

King's College is situated in the Strand, where it adjoins Somerset House. The hospital is in Portugal Street, Lincoln's Inn Fields, about five minutes' walk from the college. King's College and University College have many things in common; but one feature of King's may be noted as of some interest to parents. The religious and moral welfare of the students is a subject of special care on the part of the authorities. Throughout the winter session the principal, Dr. Wace, lectures every Friday morning on Divinity. These lectures all matriculated students of the first year are required to attend. Attendance after the first year is optional. The subject during the coming session will be the "Christian Creed and its Evidences." Moreover, the Dean of the Medical Faculty at the close of each division of the winter session and at the close of the summer session prepares a report of the attendance, general character and conduct of each student, which is forwarded to his parent or guardian after being submitted to, and signed by, the Principal. We do not stay to discuss whether this religious and moral supervision is desirable. The system that obtains has been noted, and it may be left to parents to judge for themselves whether it is a good system. The medical school at King's College is especially strong in surgery. King's College Hospital is the hospital of Sir Joseph Lister, and may thus be regarded as the home of the antiseptic system, which has changed the whole face of modern surgery. Sir Joseph Lister has just retired from active service at the hospital to become consulting surgeon, but somehow or other there is nothing in the hospital, from the operating theatre, where the Listerian system of surgery has been perfected, to the most remote ward, that does not seem to speak of Lister. The course of systematic surgery, of which Mr. Cheyne is professor, is particularly full and thorough. There are also classes of practical surgery, including minor surgery and demonstrations in surgical pathology; of operative surgery, and of clinical surgery. King's is very proud of its series of laboratories, which are extensive and complete. A special department of public health has been organised in connection with the College. King's was one of the first schools in this country to recognise the new science of bacteriology by establishing a bacteriological laboratory. Students at King's have one advantage in that there is a staff of tutors who give free instruction in chemistry, physics, medicine, and surgery. All students who are elected to resident appointments at the hospital have rooms and commons free. Unlike University College, King's has a small residential college.

The fees at King's College and University College are almost exactly the same. At University College the composition fee is £136 10s., at King's £135.

### The West London Schools.

The five schools that remain to be considered are all situated in Western London. The hospitals with which they are connected are not so large as the great charities of the East-end; but of the five, two have over 300 beds, and one (St. Mary's) has in prospect an addition of 100 beds to the 281 which it contains at present, and surely no hospital in this country that contains 300 beds can properly be described as small. Other things being equal, the school that has a healthy, pleasant situation has advantages, and undoubtedly many parents who are careful for the health and the æsthetic sensibilities of their sons show favour to such schools as St. George's and St. Mary's on this account.

### St. Mary's Hospital.

Within ten minutes' walk of the Great Western Station at Paddington stand the hospital and medical school of St. Mary's. The foundation-stone of this hospital was laid in 1847 by the late Prince Consort, and the school was opened in 1854. The hospital has been several times extended. It now contains 281 beds, and it is hoped that before very long 100 additional beds will be available in the Clarence Memorial wing, of which the foundation-stone was laid by the Prince of Wales last year. In this wing provision will be made for a residential college, for a new out-patient department, and for lying-in wards, St. Mary's will be the first general hospital in London to establish wards of this class, and the experiment will be watched with great interest. If it succeeds—and no attention to hygienic conditions and to proper isolation will be wanting to secure the best results—the advantage to midwifery students will be great. The medical school is very adequately housed in a building adjoining the hospital, and connected with it by a subway. The school buildings have recently been much extended and improved by the addition of a new library, pathological, physiological, and hygiene laboratories. and special rooms for class teaching. The museum is large and well lighted, and contains some very fine anatomical specimens and dissections. The dissecting room at the top of the building is well planned. In visiting the various laboratories one was specially struck with the completeness with which they are fitted up with all the most recent scientific appliances. The bacteriological and public health laboratories were especially noteworthy in this regard. An important feature of the work at St. Mary's is the complete system of tutorial assistance that prevails. There are special classes for the preliminary scientific examination of the London University, and, when it is desired, classes are arranged for the M.B. London and for the F.R.C.S. At the hospital there are a goodly number of resident appointments. A resident dresser is appointed by the medical superintendent once a fortnight, and receives board and residence in the hospital free of charge. Altogether St. Mary's is a thriving school, and its prosperity is well-deserved.

### St. George's Hospital.

To call the medical school at St. George's a fashionable school would not be quite just, for to many minds "fashionable" implies some things that are not desirable. Still, it is a fact that St. George's does attract students of the class whose parents are wealthy and have been able to give their sons a good education. For example, St. George's has among its students a large proportion of Oxford and Cambridge men. To what extent St. George's owes its reputation to its site is a subject for speculation; but it must be admitted that that site, at the corner of Hyde Park, is one of the finest in London. St. George's Hospital contains 356 beds, which are almost always full. These are supplemented by 100 beds at the Atkinson Morley Hospital at Wimbledon, to which chronic cases are drafted. From the student's point of view it is worthy of note that all the wards in the hospital are thrown open to students, with but few restrictions. What that means is this: At some hospitals it is the rule that students, unless they be clerks or dressers, are only admitted to the wards when they are accompanied by one of the members of the staff. At St. George's it has fortunately been found unnecessary to impose this restriction. Relying upon the gentlemanly bearing of the students, and upon their willingness to assist in maintaining the discipline of the hospital, the governors have seen their way to allow them free access to the wards at all convenient hours. A thorough system of clinical instruction is one of the features of St. George's. To obtain house office in the hospital is a prize indeed, for it implies experience of a varied and extensive kind that will prove invaluable in after life. Every three months two students are selected by competition to hold house office. Let us follow the career of one of the successful candidates: For the first six months he will be entitled to hold office as assistant in the special departments of the hospital. These include obstetric medicine and ophthalmic surgery. He will then act for three months as assistant house surgeon, and for three months thereafter as assistant house physician. At the end of this time he will be nominated by the board of the hospital for appointment as a resident house officer, which will imply his holding the position of house surgeon for six months and that of house physician for another six. A better equipment for the work of a medical practitioner than that involved in passing through the various stages of this elaborate clinical course can hardly be imagined. The school buildings are behind the hospital, a quadrangular court laid out for tennis intervening. In the arrangements of the rooms there has been a greater regard for artistic effect than in most of the London schools. The museum is extensive and admirably arranged, and there is an exceedingly complete catalogue, to the compiling of which immense care must have gone. It may not be generally known that St. George's possesses the skin of the cow from which Dr. Jenner took the first vaccine] matter. John Hunter, the great anatomist, died in St. George's Hospital. The library, the dissecting-room, the class-rooms, and laboratories were also visited. These are well equipped, and the accommodation is sufficient. St. George's has no residential college. Adjoining the school are the rooms of the students' club, which include a commodious restaurant, where wholesome meals may be obtained at moderate rates, and a reading-room. A *St. George's Hospital Gazette* was recently started, and has reached its seventh number. As we have noted, many Oxford and Cambridge men find their way to St. George's, and of its past students a large number have taken University degrees. In these examinations, as well as in the competitive examinations of the Army and Navy Medical Departments, the admirable clinical training afforded at the hospital has stood St. George's men in excellent stead.

### The Middlesex Hospital.

At the time of our visit to the Middlesex school the hospital was closed for repairs. Although the Middlesex is situated in West London, it is surrounded by a large working-class population, and the necessities of the sick poor in the vicinity make a full demand upon its 320 beds, and upon the medical charity offered in its out-patient department. The hospital was founded in 1745, but the school, which was established mainly through the exertions of Sir Charles Bell, did not come into existence until 1835. Originally the school buildings consisted of a chemical theatre, library, dissecting-room, anatomical theatre, and museum, situated in the rear of the hospital. As time went on and students grew more numerous, necessary additions were made. Middlesex is now well furnished with buildings. A noteworthy feature of the course of study at this school is that no attempt is made to teach purely science subjects, except in so far as chemistry and a modicum of biology are required under the five years' curriculum prescribed by the General Medical Council. To become entitled to sit for the M.B. of London one must have passed the preliminary examination in science of the University. In order that students may be prepared for this examination special classes are necessary, and at all the London schools, except Middlesex, these are provided. Middlesex has had the courage not to do so, but to refer students who desire to enter for the preliminary scientific examination to University College or King's College. Having taken a scientific course at one or other of these colleges, the student goes forward to the preliminary examination. This passed, he returns to Middlesex to complete his studies. For general students the composition fee is 120 guineas, but for students requiring preliminary instruction in science it is 140 guineas. But those who have paid the latter fee are thereby entitled to attend the courses of instruction in the subjects of the preliminary scientific examination held in the Faculty of Science at University College. In view of the keen competition between the London schools it required some courage on the part of Middlesex to give up these science classes, but it was a right step to take, and it would be well if the example were followed. At any rate, those who approve the position taken up by Middlesex believe that this school has led the way in a movement that will sooner or later become general. Meanwhile the school has in no respect suffered loss. It continues to enrol men who contemplate entering for University degrees, and when these come to their examinations in the subjects which they have been taught at Middlesex, they are able to hold their own with credit. With the exception that has been discussed, the course at Middlesex is as full as that at any other school. Special provision is made for dental students, of whom a large number take their general course at this hospital. Middlesex Hospital is large enough to afford every student ample opportunities for the study of disease, and there are plenty of appointments which the deserving and industrious may hope to win. The residential College is a large well-appointed building in which full provision has been made for the comfort of boarders. As at Guy's the House Physicians and House Surgeons of the hospital occupy rooms in the College.

St. Mary's, St. George's, and Middlesex, are sufficiently alike in regard to the size of their hospitals, and the character of the training that they impart, to justify their being regarded as a group by themselves.

The composition fee, covering systematic lectures and hospital practice, is for each of these three schools as follows:—

| | | | |
|---|---|---|---|
| St. George's ... ... ... ... ... | £145 | 9 | 0 |
| St. Mary's ... ... ... ... ... | 130 | 0 | 0 |
| Middlesex ... ... ... ... ... | 126 | 0 | 0 |

### Westminster and Charing Cross.

Of the general hospitals in London Westminster and Charing Cross are the smallest. Their medical schools are also small. This is not said to their disparagement. For Westminster and Charing Cross it is claimed, and with good reason, that from the students' standpoint there is an advantage in their very smallness, inasmuch as it is thereby possible to give greater attention to the individual student, to provide thorough tutorial supervision, and to have a strict care for the moral welfare of the student that is impossible in the larger schools.

### Westminster Hospital.

It is a fact of some interest in regard to Westminster Hospital, that in respect of age it stands third among the London hospitals, St. Bartholomew's and St. Thomas's being its only seniors, while Westminster is the first hospital in London that was founded by the voluntary contributions of the public. Established in 1715 in Birdcage Walk, its habitation was repeatedly changed between that year and 1834, when a resting place was found in the buildings in Broad Sanctuary, which are now furnished with over 200 beds. In the year 1834 a school of medicine was established in Westminster, but it was not till 1849 that it became connected with Westminster Hospital. The buildings in which the school is at present housed are in Caxton Street, close to the Westminster Town Hall, and at some little distance from the hospital. Erected at a cost of over £8,000, these buildings were opened in 1885. There are class-rooms, and theatre, a good dissecting-room, library, and museum already well furnished with anatomical and pathological specimens. There is a full course of study at Westminster, which includes special classes in several branches of medicine and surgery. These classes are for the most part taught in the hospital, where there are separate departments for diseases of the eye, of the ear, of the skin, of the teeth, of the throat, for those peculiar to women, and for orthopædic practice, in each of which special opportunities are offered for clinical study. At the Westminster every student is certain of getting plenty of work in dressing and clerking. "No student shall be considered as having attended the full period of hospital practice unless he have satisfactorily performed the duties of clinical clerk and dresser." Westminster School and Hospital are excellently staffed.

### Charing Cross Hospital.

Although Charing Cross comes last upon our list it must not be thought that this is in any respect a second-class school. On the contrary, it is one of the schools that has been growing in size and reputation. Anyone who will consult the staff list may satisfy himself that the hospital and school have been fortunate enough to enlist the services of a goodly number of men who have risen high in the medical world. Notwithstanding that King's, St. Thomas's, and Westminster are not far off, Charing Cross fills an important place in the hospital work of London, and helps to supply the medical and surgical needs of a densely-peopled district. Street accidents, as may readily be believed, are especially common in the neighbourhood, and the hospital is called upon to deal with a very large number of these cases. There is an arrangement in force at the hospital under which each of the surgical dressers in turn take duty as dressers for the day. The duty of this dresser, who remains in the hospital from nine a.m. to nine p.m., is, subject to the instructions he may receive from one of the house surgeons, to attend to all cases of accident that are brought in. The hospital contains 175 beds, but in addition to these the practice of the Royal Westminster Opthalmic Hospital, situated in the same buildings, with 30 beds and a large out-patient department, is free to all Charing Cross students. From 1834, when it was established, until 1881, accommodation was found for the medical school in the hospital buildings. The growth of the school having rendered better accommodation necessary, the present school was erected in Chandos Street, opposite the hospital, and connected with it by a subway. 1889 witnessed a considerable enlargement of the school buildings, designed to provide accommodation for practical instruction. The buildings are compact and well arranged. On the ground floor there are the library, the museum, with two galleries, and the students' club-room. On the first floor are chemical and physiological laboratories, the latter with places for sixty students, and a large physiology theatre. The upper floor is devoted for the most part to anatomy and pathology. The dissecting-room is well lighted from the roof, so also are the anatomical theatre and the materia medica museum. An anatomical class-room and pathological laboratory are on this floor, also the post-mortem room, which is connected by a lift with the subway to the hospital. It may be noted that every student is required to hold the appointment of pathological assistant for one month. Instruction is given in all subjects required for the diplomas of the examining bodies and for University degrees in medicine. There is a full course in Public Health, in connection with which arrangements have been made with the medical officer of health for the Strand district to give the six months' outdoor instruction for the D.P.H. In connection with clinical medicine and surgery a number of classes in special subjects are held. Clinical instruction in the diseases of the eye is given daily in the adjoining Royal Westminster Ophthalmic Hospital. Tutorial classes, which are free, are held for students who are about to present themselves for the final examination, during both winter and summer sessions. Special provisions are made for dental students. There is a students' club,

At Charing Cross and Westminster the fees are lower than they are at any of the other schools. At Charing Cross the composition fee is £115 10s., at Westminster £115.

The eleven metropolitan hospital schools have now been enumerated, but there is a twelfth which deserves a place in any account of medical education in London. It is the School of Anatomy, Physiology, and Surgery that is conducted by Mr. Thomas Cooke, M.D., F.R.C.S., assistant surgeon to the Westminster Hospital. Mr. Cooke's classes have been recognised by the University of London and other examining bodies. They are designed to meet the needs of (1) a limited number of "earnest" men who desire a thorough grounding in practical anatomy and experimental physiology; (2) of gentlemen who have been rejected at their anatomical and physiological examinations—these can get "signed up" from this school for the three or six months' supplementary work that they are now required to put in before re-examination; and (3) of qualified men who wish to obtain some of the higher qualifications, or to compete for appointments in the army, navy, and Indian medical services. Mr. Cooke is an enthusiastic teacher of large experience, whose work has been so good that it has been rewarded by recognition on the part of the examining bodies.

Very little has been said above of the scholarships and money prizes that are offered at all the London schools. Suffice it to say that each school is more or less plentifully furnished with these, a large proportion of them being entrance scholarships. The prospectuses give very full information with regard to these scholarships. This is proper enough; but one must beware of regarding the sum that is available for scholarships and exhibitions as any criterion of the efficiency of a school. Nevertheless, these prizes are a legitimate object of the student's ambition, and in these days of costly living and increasingly expensive education, he is to be reckoned fortunate who has gained one.

In our account of the medical schools of London we have avoided as far as possible the mention of names, and for this reason: that in comparing the staffs of the different schools it is so easy to play off one man of eminence against another that it is less perplexing to leave names out of count altogether. From among the men who are scattered over the London schools it would be possible to construct an ideal teaching staff second to none in the world. But the parent who has to decide upon a school for his son must take things as they are. The consoling thing is that, whatever be the choice, the metropolitan schools are all so well furnished that one cannot go wrong.

